# First-Phase Left Ventricular Ejection Fraction as an Early Sign of Left Ventricular Dysfunction in Patients with Stable Coronary Artery Disease

**DOI:** 10.3390/jcm12030868

**Published:** 2023-01-21

**Authors:** Andrzej Minczykowski, Marcin Zwanzig, Mateusz Dziarmaga, Agnieszka Rutkowska, Marek Baliński, Tomasz Krauze, Przemysław Guzik, Andrzej Wykrętowicz

**Affiliations:** Department of Cardiology-Intensive Therapy, Poznań University School of Medicine, 49 Przybyszewskiego, 60-355 Poznań, Poland

**Keywords:** ejection fraction, first-phase ejection fraction, longitudinal peak systolic strain

## Abstract

Left ventricular (LV) systolic function is often measured with echocardiography using LV ejection fraction (LVEF) or global longitudinal peak systolic strain (GLPSS). Global wasted work (GWW), global work efficiency (GWE), and first-phase ejection fraction (LVEF-1) are newer LV systolic function indices. We examined these parameters in 45 healthy individuals and 50 patients with stable coronary artery disease (CAD), normal LV contractility, and LVEF > 50%. Compared to healthy individuals, CAD patients had similar LVEF but increased GLPSS and GWW and reduced GWE and LVEF-1. The highest area under the receiver operating characteristic for detecting CAD was found for LVEF-1 (0.84; 95% CI 0.75–0.91; *p* < 0.0001), and it was significantly larger than for GLPSS (+0.166, *p* = 0.0082) and LVEF (+0.283, *p* = 00001). For LVEF-1 < 30%, the odds ratio for the presence of CAD was 22.67 (95% CI 6.47–79.44, *p* < 0.0001) in the logistic regression adjusted for age, sex, and body mass index. Finding LVEF-1 < 30% in an individual with normal LV myocardial contraction and preserved LVEF strongly suggests the presence of CAD.

## 1. Introduction

Left ventricular (LV) dysfunction is a common consequence of coronary artery disease (CAD), particularly in individuals who have experienced a myocardial infarction or have chronic critical coronary artery narrowing. Assessing LV systolic function is crucial for patients’ diagnosis, treatment, and prognosis.

The left ventricular ejection fraction (LVEF) is the most commonly used single parameter for evaluating LV function, but it has several limitations, such as reproducibility, image quality, and endocardial border definition. LVEF is a measure of global LV systolic function that can be assessed using various noninvasive methods, including two- (2D) and three-dimensional echocardiography, cardiac magnetic resonance imaging, computed tomography, and nuclear medicine imaging, such as single-photon emission computer tomography [1]. LVEF can also be estimated invasively during cardiac catheterization. However, LVEF is typically measured using 2D echocardiography in clinical practice due to its low cost and wide availability, including at the bedside.

Different methods may produce slightly different LVEF values, and the choice of method depends on the individual patient and the specific clinical context [1]. While the description of LV systolic function using LVEF is approximate, many studies have demonstrated its practicality and clinical usefulness, particularly in patients with severely compromised LV function. 

A diminished LVEF < 40% defines heart failure with reduced LVEF (HFrEF) [2]. Based on LVEF > 50% as an indicator of preserved global LV systolic function, many patients with CAD are at risk of developing heart failure (HF).

Global longitudinal peak systolic strain (GLPSS) measured using speckle tracking echocardiography can detect subtle impairment of LV systolic function [3]. Like LVEF, GLPSS reflects global LV systolic function, but it is more sensitive to LV dysfunction than LVEF and provides additional prognostic information. However, GLPSS may be afterload-dependent [4]. Recently, some new indices of LV systolic function, including the first-phase ejection fraction (LVEF-1) and myocardial work (MW), may be less dependent on afterload than LVEF and GLPSS [5,6,7].

Gu et al. [8] introduced LVEF-1 as a measure of the earliest part of left ventricular (LV) mechanical contraction related to blood ejection into the aorta. Unlike LVEF and GLPSS, LVEF-1 is not a global marker of LV systolic function. Instead, LVEF-1 is an index of the most dynamic part of LV ejection, occurring immediately after the opening of the aortic valve until the peak flow through the aortic valve. LVEF-1 represents the rapid myocardium shortening deactivation and myocardial wall stress reduction. It has been shown to predict preclinical HF in hypertension. Additionally, LVEF-1 may approximate an increased risk of adverse events in asymptomatic patients with aortic stenosis and preserved LVEF, and premature mortality in patients with COVID-19 [6,8,9,10,11,12,13,14,15,16].

Russel et al. [17] explored left ventricular pressure-strain loops and proposed several indices to approximate the global work of LV myocardium. MW generally refers to all the LV’s mechanical work performed to pump blood to the aorta. However, only some MW relates to effective blood ejection to the aorta, representing the constructive MW. The ratio of the constructive MW to the total MW measures global work efficiency (GWE) [18]. In contrast, the proportion of mechanical systolic LV work not contributing to ejection is the global wasted work (GWW) [19]. However, most wasted work appears during the late systole and early diastole, right before and after the aortic valve closure when diastole begins. The LV myocytes start isometric relaxation, followed by their lengthening, but some segments of the LV continue to shorten in the early diastole, causing a partial waste of mechanical LV contraction. Indices of MW have been studied in patients with cardiac dyssynchrony, HF, CAD, or survivors of myocardial infarction [18,20,21]. Usually, if the GWW increases, values of GWE decline.

To date, no study has directly compared LVEF and GLPSS with GWW, GWE, and LVEF-1 in CAD patients with no LV contractile abnormalities and LVEF > 50%. As such, the present investigation aims to explore this issue. For this reason, the diagnostic features of standard and new echocardiographic measures of the LV systolic function in healthy control subjects were compared to patients with established CAD and preserved LVEF. 

## 2. Materials and Methods

### 2.1. Sample Size Estimation

We used the following assumptions for sample size computation:

1. Nonparametric comparison of unpaired data with the Mann–Whitney test.
2. Statistical power set at 0.8 and two-sided alpha at 0.05.
3. As there are limited data on LVEF-1 and MW in CAD patients with normal contractility, we have used GLPSS instead as the main parameter, which might differentiate CAD patients from healthy people. Additionally, GLPSS varies between vendors of echocardiographic systems, e.g., GE, Philips, and Tomtec. In our department, we work with GE echocardiographs. Therefore, we searched for papers using the same system for GLPSS studies. Based on our own experience and the study by Tsai et al. [22] (who also used echo from GE to investigate a similar population of CAD patients without LV contractile abnormalities vs. people with no CAD (LVEF in both groups around 70%)), we input GLPSS mean ± standard deviation values for the control group (−19% ± 2.5%) and CAD patients without contractile abnormalities (−16.5% ± 4.5%).

The estimated minimum sample size was 38 people in each group (online tool at https://homepage.univie.ac.at/robin.ristl/samplesize.php?test=wilcoxon, accessed on 8 August 2022). We used data from 45 healthy people and 50 CAD patients with no LV contractile abnormalities to limit the risk of insufficient statistical power of our analyses.

### 2.2. Participants

#### 2.2.1. Healthy Volunteers

In the past (November 2018 and May 2019), we gathered healthy volunteers for the Department’s Echo Lab internal reference database for a newer echo machine (Vivid E95, GE Vingmed Ultrasound AS, Horten, Norway) purchased in October 2018. Routinely, all people enrolled in our reference database undergo a clinical evaluation, which includes brachial blood pressure measurements and standard 12-lead ECG recording before transthoracic 2D echocardiography. We used the following criteria to define participants as healthy: no known acute or chronic illness, no signs and symptoms of any disease, and not taking any chronic medication except for oral hormonal contraception in women of reproductive age. Smokers were allowed to participate. Occasional use of non-steroid anti-inflammatory drugs for occasional pain (e.g., headache) was allowed but not in the 48 h preceding ECG and echo studies. Sinus rhythm and normal findings on ECG, brachial blood pressure (<130/80 mmHg) at supine rest, and echocardiographic measurements were obligatory for all healthy people. 

For this study, we used anonymized data from our reference database from all consecutive volunteers who had available cine loops to measure LVEF-1 and MW indices. No other data selection criteria were applied, and the data were used retrospectively.

#### 2.2.2. Patients with a Stable Coronary Artery Disease

From our clinical database of patients who have undergone clinically indicated coronary angiography (between 1800 and 2000 procedures per year), we selected data from 50 consecutive CAD individuals who also had performed routine 2D echocardiography on the Vivid E95 and fulfilled the following criteria:

− Good quality images and cine loops allowing the measurement of LVEF-1 and MW in the post-processing (directly on Vivid E95 or EchoPAC platform by GE)
− No contractile abnormalities of the LV and right ventricle
− Presence of a significant (over 50%) narrowing of the lumen of at least one coronary artery. All coronary lesions were assessed by invasive angiography according to the current guidelines of the European Society of Cardiology [23,24]. Visual angiographic assessment and computer-assisted quantitative coronary analysis (QCA) using the Philips Allura Xper FD 10 software version R1.3.2 (10.2.0.10090) (Philips Medical Systems Nederland B.V., Best, The Netherlands) were performed to evaluate coronary artery stenosis.

Individuals with any clinically significant valvular disease, defined as more than mild regurgitation or stenosis, were excluded from the study.

Because the study had no experimental features and was retrospective, the local Ethics Committee waived the requirement to obtain patients’ agreement and permitted the use of data from our department’s databases. This approach is consistent with the Declaration of Helsinki for retrospective studies [25]. 

### 2.3. Clinical Examination

Peripheral arterial blood pressure was measured using an Omron 705 IT device (Omron Healthcare Co., Ltd., Kyoto, Japan). Echocardiographic analyses were performed using a Vivid E95 equipped with an M5S 3.5 MHz transducer. Standard techniques outlined by the American Society of Echocardiography were used to obtain 2D, M-mode, and Doppler measures [26]. 

Digital images were captured and stored for further offline analysis. LV end-diastolic volume (LVEDV), LV end-systolic volume (LVESV), and left ventricular ejection fraction (LVEF) were calculated in four- and two-chamber views using the biplane modified Simpson’s method. A pulsed-wave Doppler (PW) was used to measure the peak velocity of the E wave, and Doppler tissue imaging for estimation of the mitral annular early diastolic velocity (E′) in both the septal and lateral annulus, and these were averaged to calculate the E/E′ ratio. 

### 2.4. First-Phase Ejection Fraction 

LVEF-1 is calculated as the relative LV volume change from end-diastole to the time of maximal velocity of aortic flow (T1V):

LVEF-1 = (LV end-diastolic volume (LVEDV) − T1V)/LVEDV × 100%

T1V is measured using a biplane-modified Simpson’s method. The time difference between the peak of the ECG R-wave and a maximal aortic flow velocity recorded by PW gate in the LV outflow tract, close to the aortic valve leaflets, from an apical five-chamber view, were used to estimate T1V. 

### 2.5. Myocardial Work Quantification 

Echocardiographic continuous dynamic images of the LV apical four-chamber, three-chamber, and two-chamber views at a frame rate of at least 60 frames per second were obtained. Depth, sector width, and gain were optimized for adequate LV myocardium visualization. Automated Function Imaging software of the Vivid E95 ultrasound system (GE Vingmed Ultrasound AS, Horten, Norway) was used to perform speckle-tracking analysis. GLPSS and brachial blood pressure recordings measured before the echocardiography were used to quantify global MW indices through the noninvasive LV pressure-strain analysis [27]. In this method, the LV pressure-strain loop curve is constructed and then adjusted to the duration of isovolumic and ejection phases, which are defined by the timing of mitral and aortic valvular events evaluated through 2D echocardiography. Myocardial work and related indices were calculated using a specific module within the Automated Function Imaging software version 202.

### 2.6. Statistical Analysis 

Qualitative data were coded as either 0 (for women or individuals from the control group) or 1 (for men or CAD patients) and then represented as numbers and percentages. The gender distribution between the control group and CAD patients was compared using the Fisher exact test. As most continuous data did not have a normal distribution (according to the D’Agostino–Pearson normality test), results are summarized as medians and the 25th and 75th percentiles (IQR). 

Comparisons between healthy individuals and CAD patients were made using the unpaired Mann–Whitney test. For exploratory purposes, only LV systolic function parameters that differed significantly were compared using analysis of covariance (ANCOVA), a priori adjusted for subjects’ age, gender, and body mass index (BMI). Only ANCOVA models with a normal distribution of residuals (as determined by the Cook’s and Mahalanobis distances) were accepted and presented, even if the original covariates did not have a normal distribution. 

Nonparametric Spearman tests were performed to establish correlations between LVEF or GLPSS and newer LV systolic function indices. Multivariate linear regression was analyzed to study the association of LV systolic function indices with sex, gender, blood pressure, heart rate, and LV mass index. Receiver operating characteristic (ROC) characteristics with the area under the curve (AUC) were computed for various measures of LV systolic function to determine their potential for differentiating control subjects from CAD patients.

Additionally, the obtained ROC curves were compared using the method of DeLong et al. [28] to test whether newer indices of LV systolic function have significantly larger AUCs than LVEF and GLPSS. For all parameters with AUCs significantly departing from 0.5, the optimum cut-off points with corresponding sensitivity, specificity, positive predictive value, negative predictive value, and accuracy were calculated using the maximum value of Youden’s index. Finally, the cut-offs of all LV systolic function indices were tested first in the unadjusted and then in a priori adjusted models for participants’ age, gender, and BMI using univariate logistic regression; the purpose of this was to obtain the odds ratio (OR) with the corresponding 95% confidence intervals (CI). 

As some critical parameters, such as gender distribution and BMI, were not comparable between the control and CAD groups, we used sex for the case-control matching. The results of comparisons of continuous data for the 31 case-control matched pairs by the paired Wilcoxon test are shown in the supplementary material.

A *p*-value < 0.05 was considered statistically significant. MedCalc^®^ Statistical Software version 20.110 (MedCalc Software Ltd., Ostend, Belgium; 2022) and PQStat Software version 1.8.4.136 (PQStat Software, Poznań, Poland; 2022) were used for statistical analyses. 

## 3. Results

### 3.1. Clinical Characteristics 

The proportion of men in the control group was significantly lower than in the CAD group (31.37% vs. 68.63%; *p* = 0.0008). Fifteen (30%) healthy individuals and 19 (38%) CAD patients were active smokers. Following the inclusion criteria, no healthy subjects had any chronic disease or took pharmaceutical agents regularly (except five women on regular oral contraception). 

In the CAD group, 11 (22%) had a previous myocardial infarction, 14 (28%) had undergone percutaneous coronary intervention (PCI) in the past, one (2%) had undergone coronary artery bypass grafting (CABG), 42 (84%) had hypertension and all were well controlled, 11 (22%) had type 2 diabetes, and three (6%) had suffered either a transient ischemic attack (TIA) or ischemic stroke in the past. Thirty-two (64%) patients had single-vessel CAD, 16 (32%) had two-vessel CAD, and 2 (4%) had three-vessel CAD. Among them were three patients with a significant narrowing of the left main coronary artery (one patient in the single-, another in the two-, and the third in the three-vessel disease subgroups). 

All CAD patients were taking at least one antiplatelet drug and lipid-lowering therapy (a statin or ezetimibe), 36 (72%) were taking either an angiotensin-converting enzyme inhibitor or an angiotensin-2 receptor blocker, 34 (68%) were on a beta-blocker, 16 (32%) on a calcium antagonist, 3 (6%) on a mineralocorticoid antagonist, and 11 (22%) were taking a diuretic. 

Table 1 summarizes the statistics of the continuous variables and the comparison between healthy controls and CAD patients, showing that the CAD patients had lower BMI, LV GLPSS, GWE, and LVEF-1, but higher GWW. The statistical power for the GLPSS comparison between the control and CAD groups was 0.8838.

### 3.2. Adjusted Comparison of LV Systolic Function

Table 2 summarizes the ANCOVA results adjusted for age, gender, and BMI, comparing healthy individuals with CAD patients. After adjustment, the ANCOVA showed that CAD patients had significantly worse GLPSS, GWE, LVEF-1, and increased GWW.

### 3.3. Correlation Analysis of the Standard and Newer Indices of LV Systolic Function

Table 3 shows the Spearman correlations between GLPSS or LVEF and GWE, GWW, and LVEF-1 for the pooled data of healthy controls and CAD patients. LVEF by Simpson’s method only significantly correlated with GWE; those with higher LVEF also had better GWE. However, GLPSS was significantly associated with all newer LV systolic function indices. All participants had a better GLPSS associated with higher GWE and LVEF-1 and lower GWW.

### 3.4. Association between the LV Systolic Function and Other Clinical Parameters

Table 4 summarizes the findings of the multivariate linear regression exploring the potential associations between LV systolic function indices and sex, gender, HR, SBP, DBP, and LVMI. None of the LV systolic function parameters was affected by either sex or age of the studied participants. LVEF was not associated with blood pressure, HR, or LVMI. GLPSS was positively linked with DBP, HR, and LVMI. GWW was positively associated with SBP, HR, and LVMI. GWE was negatively correlated with HR and LVMI. LVEF-1 was negatively associated only with LVMI.

### 3.5. The Area under the Curve Analysis

Only LV systolic function indices that were significantly different in the ANCOVA were subjected to ROC analysis. Table 5 summarizes the AUC values from the ROC analysis, the cut-offs, and their corresponding specificity, sensitivity, positive predictive value, negative predictive value, and accuracy for standard (LVEF and GLPSS) and newer (GWW, GWE, and LVEF-1) LV systolic function indices. The table illustrates that, except for LVEF, all parameters had the potential to differentiate CAD patients from healthy controls. However, the highest AUC was for LVEF-1, and since the AUC for LVEF was not significantly different from 0.5, the cut-off value and the descriptors of its diagnostic properties were unavailable.

**Figure 1:**
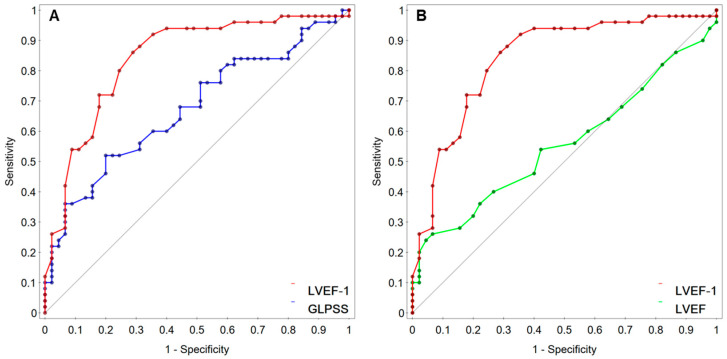
Comparisons of AUCs (area under the curve) for LVEF-1 (first-phase ejection fraction) with GLPSS (global longitudinal peak systolic strain) (**A**) or LVEF (left ventricular ejection fraction) (**B**).

Table 6 summarizes the paired comparisons of the AUC values between LVEF or GLPSS and GWE, GWW, and LVEF-1. LVEF-1 had significantly larger AUCs than LVEF (over 0.28) and GLPSS (nearly 0.17), as shown in Figure 1. However, neither GWE nor GWW had significantly larger AUCs than GLPSS and LVEF.

### 3.6. Odds Ratio for Differentiating CAD Patients with Normal LVEF from Healthy People

The estimated cut-off values for univariate logistic regression analysis were either unadjusted or adjusted for participants’ age, gender, and body mass index. In both analyses, abnormal values of all LV systolic function indices were significantly associated with an increased odds ratio for CAD. LVEF-1 outperformed all remaining parameters, particularly in the adjusted regression logistic models, with participants exhibiting LVEF-1 < 30% being 22 times more likely to have CAD than individuals with higher LVEF-1 (Table 7).

## 4. Discussion

The newer measures of LV systolic function significantly differ between patients with stable CAD and normal LVEF and healthy individuals. Regardless of the comparable LVEF in both groups, GLPSS, GWW, GWE, and LVEF-1 indicate poorer LV systolic function in CAD patients. Larger LV mass correlates with worse systolic function, which also declines (except for LVEF-1) if heart rate and blood pressure increase. LVEF-1 outperforms the diagnostic properties of other LV systolic function measures—people with reduced LVEF-1 < 30% are at the highest risk for CAD regardless of age, gender, and BMI. 

An impaired LV systolic function with LVEF > 50% was demonstrated with GLPSS in studies on patients with aortic stenosis, hypertension complicated by HF with diastolic dysfunction, or those treated with certain chemotherapeutics [29,30,31,32,33]. Here, we present that GLPSS is also worse in patients with CAD and LVEF > 50% than in healthy subjects. This observation deserves some comments. 

GLPSS is a direct measure of the LV walls’ deformation during systole. On the other hand, LVEF quantifies the relative contribution of the blood pumped from the LV (i.e., stroke volume) to the overall LV end-diastolic volume. Although LVEF does not reflect myocardial contractions and deformation, it is an indirect measure of LV systolic function. 

A normal LVEF provides approximate information that the LV global pumping function is preserved. Nevertheless, an increased GLPSS detects some early abnormalities in myocardial deformation. In CAD patients with no visible contractile impairment and normal LVEF, deformation abnormalities could be caused by active myocardial ischemia. However, our patients had stable CAD with no angina during the examination. Long-term consequences of chronic CAD (e.g., myocardial fibrosis) might contribute to this finding. We also show that GLPSS increases with higher DBP, HR, and LVMI. Our CAD patients had significantly larger LVMI, whereas their HR and blood pressure were comparable to healthy people.

LVEF-1 measures the early systolic shortening of cardiomyocytes during the most dynamic phase of LV contraction, which is associated with blood ejection. At this time, the highest myocardial wall stress is generated. As Gu et al. [8] suggested, a reduced LVEF-1 may result from delayed shortening or deactivation of the LV myocardium. To preserve global LVEF, the myocardial contraction (i.e., the shortening of cardiomyocytes) may last longer and sometimes exceed the systole duration. As a result, some LV segments continue their contraction early in the diastole, leading to wasted and less effective myocardial work. Our findings confirm this reasoning, as we have observed that, compared to healthy controls, GWW (global wall work) increases while GWE (global wall efficiency) declines in CAD patients.

Our study found that LVMI negatively impacts all newer LV systolic function indices. Wasted MW increased, while GWE and LVEF-1 decreased with a larger LV mass, which alters LV geometry, raises myocardial wall stress, and changes LV systolic performance. Higher GWW appears to be a consequence of LVEF-1 reduction. At normal LVEF, the LV pumping function is preserved at the expense of diastolic function when some myocardial segments continue their contraction. In our study, LVEF-1 and GWW were negatively correlated (rho = −0.34; *p* = 0.0001—data not presented in Results). As shown by Gu et al. [8] and Chirinos et al. [34], people with hypertension and reduced LVEF-1 or increased late systolic myocardial loading gradually develop diastolic dysfunction—isovolumetric relaxation is impaired, LV diastolic pressure increases, and the left atrium dilates and shows abnormal function [8,34]. In CAD, diastolic dysfunction is a common finding, usually preceding LV systolic impairment [35].

Compared to other studies on LVEF-1 and MW indices, we demonstrate that LVEF-1 is also a sensitive marker of subclinical LV dysfunction in CAD patients with normal myocardial contractility and preserved LVEF. We also demonstrate that LVEF-1 < 30% is significantly associated with a substantial risk of CAD, regardless of the patient’s age, gender, and BMI.

### 4.1. Study Limitations 

This was a retrospective study using two distinct sets of data. The control group consisted of clinical data from consecutive healthy volunteers who underwent a 2D transthoracic echocardiography for a reference database for a newer echocardiograph (Vivid E95). The CAD group consisted of consecutive patients who met certain criteria, including having no contractile abnormalities, LVEF > 50%, and available 2D echocardiography recordings on the same echo machine. Although the minimum sample size was estimated to be 38 people per group, data from 45 healthy individuals and 50 patients with CAD were used. 

Despite the expected clinical differences between healthy individuals and those with CAD, the sex distribution and BMI significantly varied between the two groups. This resulted from using consecutive data from healthy people and CAD patients. Therefore, an additional case-control matching based on sex was performed (results shown in the Supplementary Material). Paired comparisons (Wilcoxon test) confirmed that GLPSS, GWW, GWE, and LVEF-1 were significantly worse in CAD patients than in healthy individuals (Table S1). Moreover, the thresholds defined for the unmatched participants were also applied after sex-based case-control matching. GLPSS > −17%, GWE < 93%, GWW > 123 mmHg%, and LVEF-1 < 30% were all associated with an increased odds ratio for CAD, even in the analysis adjusted for age and BMI (Table S2).

The sample size for this study was relatively small, but the statistical power for the Mann–Whitney test was strong (0.8848) for GLPSS. Additionally, for LVEF-1, the statistical power was even stronger (0.9998). 

Our CAD patients share several common features, such as having stable CAD, no LV contractility impairment, and normal LVEF > 50%. However, this group consists of quite heterogeneous patients. Some of them have survived MI, TIA, or stroke, undergone PCI (one person had CABG) in the past, and have diabetes. Among them are single-, two-, and three-vessel CAD patients. All of these factors might be considered bias factors. Unfortunately, it is impossible to control and adjust for all of these factors in the multivariate analyses with a group of 50 people and obtain reliable results. However, we acknowledge the presence of potential biases and that their effects should be explored in more detail in a larger CAD group.

It is important to note that our findings may not be applicable to other cardiac patients, as the study specifically focused on the diagnostic properties of newer LV systolic function indices for a specific subpopulation of CAD patients (stable with normal contractility and LVEF > 50%). Additionally, as the study population was predominantly Polish and of Caucasian race, the results may not be generalizable to other racial groups.

Finally, this study utilized 2D echocardiography to measure cardiac structure and function. While other imaging modalities such as 3D echocardiography, cardiac magnetic resonance, or cardiac computed tomography may be more accurate, they also have limitations (specific clinical indications, longer examination times, and higher costs). Additionally, LVEF-1 and MW indices analyzed in this study were initially developed for use with 2D echocardiography. 

### 4.2. Perspectives and Conclusions 

Overall, we demonstrate that GLPSS, GWE, GWW, and particularly LVEF-1 help distinguish subjects with CAD and normal LVEF from healthy people. LVEF-1 focuses mainly on the early part of the LV’s blood ejection, while GWW partially covers the early diastole. The newer echocardiographic indices emphasize the importance of going beyond global LV systolic function analysis and using other parameters that better explore other phases of LV contraction and possibly the early diastole. 

It is unclear which pharmaceutical agents may target the early ejection or early diastole, but those interacting with ATP metabolism and mitochondria are the most promising. Drugs acting at this time might and targeting cellular and mitochondrial energy production might significantly improve the treatment of CAD and possibly prevent its progression to HF. 

In summary, contemporary markers of LV systolic function aid in assessing subjects with CAD and normal LVEF; indeed, GWW, GWE, and LVEF-1 demonstrate better functional phenotyping of CAD patients with normal LVEF but early signs of systolic dysfunction.

## Figures and Tables

**Table 1:** Comparisons of continuous data between control subjects and CAD patients (median (IQR)) (Mann–Whitney test).

Parameter	Control Group	CAD Patients	*p*-Value
Age, years	62.00 (59.00–65.00)	63.00 (60.00–65.00)	0.9671
BMI, kg/m^2^	23.62 (20.71–25.43)	21.87 (19.05–23.03)	0.0022
SBP, mmHg	124.00 (115.75–132.50)	128.50 (120.00–137.00)	0.0757
DBP, mmHg	75.00 (67.00–81.00)	76.00 (70.00–83.75)	0.2679
HR, bpm	68.00 (63.00–72.00)	67.00 (60.00–77.50)	0.9405
RVd, mm	28.00 (26.00–29.00)	28.00 (26.00–30.00)	0.2109
IVSd, mm	10.00 (10.00–11.00)	12.00 (11.00–13.00)	<0.0001
LVEDd, mm	43.00 (39.75–46.25)	47.29 (41.00–51.00)	0.0063
LVEDdI, mm/m^2^	24.21 (22.50–26.01)	24.12 (21.78–26.78)	0.9139
LVMI, g/m^2^	85.90 (71.15–99.08)	107.70 (87.10–131.90)	<0.0001
E/A	2.51 (1.97–3.03)	2.21 (1.67–2.87)	0.0987
E/E′	6.90 (6.10–9.30)	7.75 (6.63–9.68)	0.2271
LVEF, %	63.00 (60.00–67.00)	62.00 (56.25–67.75)	0.3507
GLPSS, %	−18.10 (−20.30–−17.30)	−17.00 (−18.30–−15.35)	0.0035
GWW, mmHg%	95.00 (72.00–121.00)	137.00 (79.00–185.25)	0.0258
GWE, %	94.00 (93.00–96.00)	93.00 (88.00–94.00)	0.0123
LVEF-1, %	37.00 (30.00–42.00)	21.00 (17.25–29.00)	<0.0001

Abbreviations: BMI—body mass index, DBP—diastolic blood pressure, E/A—E to A waves ratio, E/E′—E to E′ ratio, GLPSS—global longitudinal peak systolic strain, GWE—global work efficiency, GWW—global wasted work, HR—heart rate, IVSd—end-diastolic thickness of the intraventricular septum, LVEF—left ventricular ejection fraction, LVEF-1—the first-phase left ventricular ejection fraction, LVEDd—left ventricular end-diastolic diameter, LVEDdI—LVEDd normalized to body surface area, LVMI—left ventricular mass index, RVd—right ventricular end-diastolic diameter, SBP—systolic blood pressure.

**Table 2:** ANCOVA of LV systolic function indices, which differed significantly in the unpaired tests (Mann–Whitney) between healthy controls and CAD patients. ANCOVA was adjusted for age, gender, and BMI, and the results are shown as the estimated marginal means (EMM) and standard error (SE).

Parameter	Control Group	CAD Patients	*p*-Value
GLPSS, EMM (SE)	−18.39 (0.47)	−16.64 (0.45)	0.0105
GWE, %, EMM (SE)	93.40 (0.77)	90.90 (0.73)	0.0255
GWW, mmHg%, EMM (SE)	109.00 (13.20)	153.96 (12.48)	0.0187
LVEF-1, %, EMM (SE)	36.65 (1.45)	23.26 (1.37)	<0.0001

Abbreviations: EMM—estimated marginal mean, GLPSS—global longitudinal strain, GWE—global work efficiency, GWW—global work wasted, LVEF-1—first-phase left ventricular ejection fraction, SE—standard error.

**Table 3:** Correlation analysis of the standard and newer indices of LV systolic function.

	GLPSS		LVEF	
	Rho	*p*-Value	Rho	*p*-Value
GWW	0.36	0.0003	−0.18	0.0753
GWE	−0.54	<0.0001	0.26	0.0119
LVEF-1	−0.35	0.0005	0.18	0.0899

Abbreviations: GLPSS—global longitudinal strain, GWE—global work efficiency, GWW—global work wasted, LVEF—left ventricular ejection fraction, LVEF-1—the first-phase left ventricular ejection fraction, rho—Spearman coefficient of variation.

**Table 4:** Multivariate linear regression for the association of LV systolic function parameters with sex, gender, heart rate, blood pressure and LV mass index.

	LVEF		GLPSS		GWW		GWE		LVEF-1	
	b Coeff.	*p*-Value	B Coeff.	*p*-Value	b Coeff.	*p*-Value	B Coeff.	*p*-Value	b Coeff.	*p*-Value
intercept	65.18	<0.0001	−23.72	<0.0001	−205.38	0.1962	106.81	<0.0001	40.60	0.0757
Sex	−1.97	0.1631	−0.49	0.4292	−35.11	0.0539	1.54	0.1183	1.10	0.6699
Age	0.15	0.3426	−0.09	0.1608	−2.19	0.2646	0.13	0.2245	0.24	0.3888
SBP	−0.02	0.7877	−0.01	0.5753	2.58	0.0009	−0.04	0.3657	−0.19	0.0756
DBP	−0.08	0.2210	0.06	0.0256	−0.74	0.3748	−0.02	0.6275	0.12	0.3265
HR	0.00	0.9610	0.06	0.0412	2.36	0.0043	−0.12	0.0075	−0.02	0.8657
LVMI	−0.03	0.1791	0.05	<0.0001	0.60	0.0254	−0.09	<0.0001	−0.09	0.0141

Abbreviations: b coeff.—b coefficient (slope) from the regression model, DBP—diastolic blood pressure, GLPSS—global longitudinal strain, GWE—global work efficiency, GWW—global work wasted, LVEF—left ventricular ejection fraction, LVEF-1—the first-phase left ventricular ejection fraction, LVMI—LV mass index, SBP—systolic blood pressure.

**Table 5:** AUC for standard (LVEF and GLPSS) and newer (GWW, GWE, and LVEF-1) LV systolic function indices to differentiate CAD patients from healthy controls.

Variable	AUC	95% CI	*p*-Value	Cut-Off Value	Sensitivity	Specificity	PPV	NPV	Accuracy
LVEF	0.56	0.44–0.67	0.3496	NA	NA	NA	NA	NA	NA
GLPSS	0.67	0.57–0.77	0.0016	>−17	52.00	80.00	74.29	60.00	65.26
GWE	0.65	0.54–0.74	0.0085	<93%	62.00	62.22	64.58	59.57	62.11
GWW	0.63	0.59–0.73	0.0221	>123 mmHg%	56.00	75.56	71.79	60.71	65.26
LVEF-1	0.84	0.75–0.91	<0.0001	<30%	86.00	71.11	76.79	82.05	78.95

Abbreviations: AUC—area under the curve, CI—confidence interval, GLPSS—global longitudinal strain, GWE—global work efficiency, GWW—global work wasted, LVEF—left ventricular ejection fraction, LVEF-1—the first-phase left ventricular ejection fraction, NA—not available, NPV—negative predictive value, PPV—positive predictive value.

**Table 6:** Differences in the AUCs between LVEF by the Simpson’s method or GLPSS and GWW, GWE and LVEF-1.

	GLPSS Compared to			LVEF Compared to		
	AUCs Differences	95% CI	*p*-Value	AUCs Differences	95% CI	*p*-Value
GWE	0.0258	−0.0864–0.138	0.6525	0.0916	−0.0500–0.233	0.2048
GWW	0.0413	−0.0905–0.173	0.5388	0.076	−0.0735–0.226	0.3191
LVEF-1	0.166	0.0429–0.289	0.0082	0.283	0.149–0.417	0.0001

Abbreviations: AUC—area under the curve, CI—confidence interval, GLPSS—global longitudinal strain, GWE—global work efficiency, GWW—global work wasted, LVEF—left ventricular ejection fraction, LVEF-1—the first-phase left ventricular ejection fraction.

**Table 7:** Univariate logistic regression for the risk of CAD with normal LV contractility and LVEF > 50% unadjusted and adjusted for participants’ age, gender, and body mass index.

	Unadjusted			Adjusted		
Variable	OR	95% CI	*p*-Value	OR	95% CI	*p*-Value
GLPSS > −17	3.41	1.36–8.53	0.0089	2.99	1.12–8.00	0.0288
GWE ≤ 93%	2.69	1.17–6.16	0.0196	2.63	1.07–6.44	0.0343
GWW > 123 mmHg%	3.63	1.51–8.73	0.0040	3.94	1.52–10.23	0.0048
LVEF-1 < 30%	15.12	5.42–42.21	<0.0001	22.67	6.47–79.44	<0.0001

Abbreviations: CI—confidence interval, GLPSS—global longitudinal strain, GWE—global work efficiency, GWW—global work wasted, LVEF-1—first-phase left ventricular ejection fraction, OR—odds ratio.

## Data Availability

Not applicable.

## Supplementary Materials

The following supporting information can be downloaded at: https://www.mdpi.com/article/10.3390/jcm12030868/s1, Table S1: Comparisons of continuous data between control subjects and CAD patients (median (IQR)) after sex-based matching for the paired case-control comparison (Wilcoxon test); Table S2: Univariate logistic regression unadjusted and adjusted for participants’ age, gender and body mass index.
