# Supplementary material for: First-Phase Left Ventricular Ejection Fraction as an Early Sign of Left Ventricular Dysfunction in Patients with Stable Coronary Artery Disease
Source: J Clin Med. 2023 Jan 21;12(3):868. doi: 10.3390/jcm12030868 (PMC9918081; doi:10.3390/jcm12030868)
Supplement: Supplementary file 1 [file jcm-12-00868-s001.zip › jcm-2062781-supplementary.pdf]

Journal of
Clinical Medicine

MDPI

Supplementary Data

Clinical Characteristics

We used sex-based case-control matching to balance the control individuals with CAD patients (PQStat, Poland) and obtained 31 balanced pairs. There are 16 men and 15 women in each group. BMI is now comparable between control subjects and CAD patients, and the median age is not significantly different. The main findings are similar to those with unmatched data, i.e., CAD patients have significantly thicker IVSd (and higher LVMI), GLPSS, GWW, and lower GWE and LVEF-1. Of note, LVEF-1 appears to be similar before and after matching in both the control group (37.00% [30.00–42.00%] vs. 38.00% [32.50–44.75%], respectively) and CAD patients (21.00% [17.25–29.00%] vs. 21.00% [16.25–28.50%]).

The statistical power of this approach for GLPSS is 0.7574. However, for LVEF-1, it is 0.9936.

Table S1. Comparisons of continuous data between control subjects and CAD patients (median (IQR)) after sex-based matching for the paired case-control comparison (Wilcoxon test).

Parameter	Control Group		CAD Patients		*p*-Value
	median	IQR	median	IQR	
Age, years	60.00	59.00–62.00	64.00	61.00–66.00	0.0817
BMI, kg/m^2	22.31	19.68–24.22	22.21	20.77–23.78	0.2476
SBP, mmHg	122.00	113.00–132.00	127.00	117.75–137.75	0.2094
DBP, mmHg	75.00	66.25–82.00	75.00	70.00–82.75	0.5506
HR, bpm	66.00	62.25–72.00	67.00	60.00–78.00	0.6359
RVd, mm	28.00	26.00–29.00	28.00	26.25–29.75	0.7532
IVSd, mm	10.00	9.25–11.00	12.00	11.00–13.00	0.0004
LVEDd, mm	43.00	39.00–48.00	45.00	40.00–49.75	0.2424
LVEDdI, mm/m^2	23.81	21.93–25.48	24.49	21.48–26.89	0.4217
LVMI, g/m^2	85.50	70.83–98.73	97.70	86.05–118.60	0.002
E/A	2.40	1.70–2.88	2.30	1.74–2.93	0.3519
E/E'	6.70	5.80–8.25	7.40	6.10–9.45	0.3176
LVEF, %	63.00	60.00–68.00	66.00	59.50–68.00	0.3632
GLPSS, %	−18.70	−20.25–−17.30	−17.40	−18.30–−15.83	0.0086
GWW, mmHg%	102.00	63.75–130.75	150.00	78.00–177.75	0.0378
GWE, %	94.00	93.00–96.00	93.00	89.50–94.00	0.0327
LVEF-1, %	38.00	32.50–44.75	21.00	16.25–28.50	0.0006

Abbreviations: BMI—body mass index, DBP—diastolic blood pressure, E/A—E to A waves ratio, E/E′—E to E′ ratio, GLPSS—global longitudinal peak systolic strain, GWE—global work efficiency, GWW—global wasted work, HR—heart rate, IVSd—end-diastolic thickness of the intraventricular septum, LVEF—left ventricular ejection fraction, LVEF-1—the first-phase left ventricular ejection fraction, LVEDd—left ventricular end-diastolic diameter, LVEDdI—LVEDd normalized to body surface area, LVMI—left ventricular mass index, RVd—right ventricular end-diastolic diameter, SBP—systolic blood pressure.

The sex-based matching confirms that GLPSS, LVEF-1, and GWE are significantly lower, while GWW is higher in CAD patients with no contractile abnormalities and normal LVEF compared to healthy people of similar age and equivalent BMI. It suggests that the earliest and most dynamic phase of LV ejection and myocardial wall shortening deactivation are impaired. Additionally, a higher wasted myocardial work in CAD patients implies that some LV segments continue their contraction in the early diastole after aortic valve closure. This wasted myocardial work reduces global myocardial work efficiency.

In summary, we demonstrate that regardless of normal LVEF and no visible contractile abnormalities in CAD patients, they have significant LV systolic dysfunction, which starts early in systole and continues through the end of systole and the beginning of diastole. LVEF-1 and strain-based indices of LV systolic function, i.e., GLPSS, GWE, and GWW, appear more sensitive in detecting LV systolic dysfunction than LVEF. Whether these newer indices might be used to redefine systolic dysfunction in cardiac patients should be further investigated in more extensive and prospective studies.

The Odds Ratio for Differentiating CAD Patients with Normal LVEF from Healthy People in a Sex-Matched Case-Control Analysis

Using the thresholds established for the original group of 45 healthy individuals and 50 CAD patients (see Table 4), we repeated the univariate logistic regression, both unadjusted and adjusted for age and BMI. Since the case-control group was matched by sex, it was not necessary to make additional adjustments for the participants' gender.

The unadjusted and adjusted logistic regressions show that abnormal values of GLPSS, GWE, GWW, and particularly LVEF-1 are significantly associated with CAD with normal LVEF (Table S2). The odds ratios for CAD in the adjusted logistic regression are higher after matching compared to the original analysis (Table 6 of the main paper). Additionally, it demonstrates that the thresholds for LV systolic dysfunction indices (such as GLPSS, GWE, and GWW) effectively differentiate CAD patients from healthy individuals, even when the two groups are matched by gender.

Table S2. Univariate logistic regression unadjusted and adjusted for participants' age, gender and body mass index.

	**Unadjusted**			**Adjusted**		
**Variable**	**OR**	**95% CI**	***p*-Value**	**OR**	**95% CI**	***p*-Value**
GLPSS > −17	3.43	1.10–10.70	0.0337	5.17	1.42–18.69	0.0123
GWE ≤ 93%	3.33	1.17–9.44	0.0241	3.22	1.10–9.47	0.0335
GWW > 123 mmHg%	4.55	1.54–13.42	0.0060	4.50	1.47–13.77	0.0084
LVEF-1 < 30%	14.28	4.28–48.67	<0.0001	24.32	5.50–107.49	<0.0001

Abbreviations: CI—confidence interval, GLPSS—global longitudinal strain, GWE—global work efficiency, GWW—global work wasted, LVEF-1—first-phase left ventricular ejection fraction, OR–odds ratio.

As in the original analysis, LVEF-1 outperformed the other LV systolic function parameters. If a person with normal LV contractility and LVEF shows a reduced value of LVEF-1 < 30%, the odds ratio for the presence of at least one significant lumen narrowing of the coronary artery exceeds 24.
